# Clinical presentation of acute appendicitis in adults at the Chris Hani Baragwanath academic hospital

**DOI:** 10.1186/1865-1380-7-12

**Published:** 2014-02-17

**Authors:** Richard Nshuti, Deirdré Kruger, Thifheli E Luvhengo

**Affiliations:** 1 Department of Surgery, Chris Hani Baragwanath Academic Hospital and University of Witwatersrand, Witwatersrand, South Africa

**Keywords:** Acute appendicitis, Complications, Delayed presentation, Negative appendicectomy

## Abstract

**Background:**

Acute appendicitis is the most common surgical abdominal emergency. Delayed treatment increases the incidence of complications. The aim of this study was to investigate the presentation, incidence, and predictors of complications, and histological findings in adult patients with clinical diagnosis of acute appendicitis.

**Methods:**

The study was a prospective observational study and included patients aged 12 years and older diagnosed with acute appendicitis. Data collected included demographic data, clinical presentation, duration of symptoms and reasons for presentation delay, diagnostic investigations, operative and histology findings, length of hospital stay, and mortality.

**Results:**

A total of 146 patients were admitted with a mean age of 26 years (SD = 12 years). The male to female ratio was 1.6:1. Predominant presenting symptoms were right iliac fossa pain (95%), nausea (80%), and vomiting (73%), with 63% of patients presenting 2 days after onset of symptoms. Fever was present in 15% and only 31% of patients gave a typical history of acute appendicitis of vague peri-umbilical pain. The negative predictive values of white cell count and C-reactive protein for acute appendicitis were 28% and 50%, respectively. Sensitivity of the ultrasound to detect acute appendicitis was 60% with a negative predictive value of 31%; 30% of patients had complicated appendicitis. Histology results showed a normal appendix in 11% of patients. The 30-day mortality rate was 1.4%.

**Conclusions:**

Patients with acute appendicitis rarely present with a typical history of vague peri-umbilical pain. The negative predictive values of both white cell count and ultrasound proved that neither of these measurements was accurate in the diagnosis of acute appendicitis. Most of our patients with complicated disease present late, with the most common reasons for this delay being lack of access to a medical clinics and prior treatment by general practitioners. Complications were higher in males and in those aged 45 years and above.

## Background

Appendicectomy is the most common emergency surgical procedure worldwide. About 8% of people in Western countries will have appendicitis during their lifetime, and the incidence in the UK is about 52 per 100,000 population. However, in South Africa, the incidence is estimated to be less than 9 per 100,000. The peak incidence of acute appendicitis is between 10 and 30 years of age [1,2].

The diagnosis of acute appendicitis is mainly clinical and presentation of acute appendicitis may be typical or atypical. Typical presentation starts with vague peri-umbilical pain for several hours, which later migrates to the right iliac fossa (RIF), associated with lack of appetite, nausea, or vomiting. Atypical histories lack this typical progression and may include pain in the right lower quadrant as an initial symptom [3].

If left untreated, acute appendicitis may lead to complications, leading to inflammatory mass, appendix abscess, or rupture, with generalized peritonitis. Diagnosis of complicated acute appendicitis is clinically supplemented by ultrasound or CT scan [4,5]. However, it is common in practice to admit and observe patients with an uncertain diagnosis and to delay their surgery until the diagnosis is more definite in order to reduce the negative appendicectomy rate. Pre-admission delay on the part of the patient and post-admission delay by the surgeon are responsible for combined delay in diagnosis and definitive management [6-8].

## Methods

This was a prospective observational study of patients 12 years and older (as 12 years is a lower age cut-off for admission), diagnosed and treated for acute appendicitis at the Chris Hani Baragwanath Academic Hospital (CHBAH) from May 1^st^ 2011 to October 31^st^ 2011.

Patients’ files were reviewed on admission and after discharge. Data retrieved included patients’ demographics, clinical presentation, and duration of symptoms before presentation to the hospital, results of diagnostic investigations and evidence of complicated disease at presentation, length of hospital stay, intensive care unit (ICU) admission, negative appendicectomy, and mortality rate.

Sensitivity, specificity, positive predictive value (PPV) and negative predictive value (NPV) of diagnostic investigations were calculated. An Excel sheet was used for data collection and Statistica was used for statistical analysis.

Permission to conduct the study was received from the Human Ethics Committee of the University of Witwatersrand and Research Review Board of the CHBAH.

## Results

**Figure 1:**
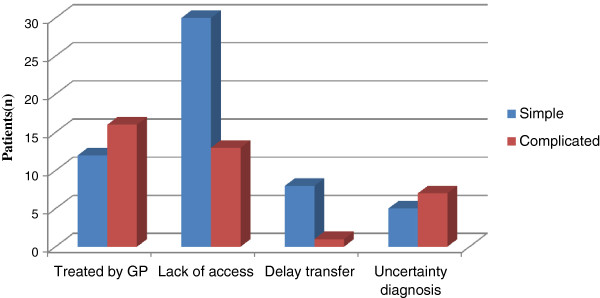
Reasons for delays compared to the occurrence of complicated appendicitis.

A total of 146 patients were diagnosed with acute appendicitis. The male to female ratio was 1.6:1 and their mean age was 26 years (SD = 12 years). The duration of symptoms was 4.5 days (SD = 4 days) and 63% of the patients presented more than two days after the onset of symptoms. Overall, the complicated appendicitis rate was 30%, with the most common reason for delay in presentation being a lack of access to hospitals or clinics and to information (29%), and prior treatment by general practitioners (19%) (Figure 1).

**Figure 2:**
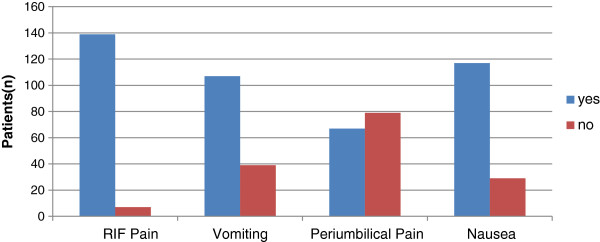
Signs and symptoms.

Common presenting symptoms were RIF pain (95%), vomiting (73%), and 31% had a typical acute appendicitis presentation and 80% had nausea (Figure 2).

The following investigations were undertaken: white cell count (WCC) in 95%, C-reactive protein (CRP) in 89%, abdominal ultrasound in 40%, CT scan in 6%, and diagnostic laparoscopy in 7% of the 146 patients included in this study. The median WCC and CRP were 11.5 (8.7–15.4) and 80.5 (30.3–171.3), respectively. The sensitivity, specificity, PPV, and NPV percentages of all investigations were as illustrated in Table 1.

**Table 1:** Results of clinical findings and diagnostic investigations in all patients

**Investigation**	**Sensitivity (%)**	**Specificity (%)**	**PPV (%)**	**NPV (%)**
Fever (n = 146)	18	83	95	5
WCC (n = 139)	48	75	84	28
CRP (n = 135)	92.5	24	80	50
Ultrasound (n = 60)	60	66	89	31
CT Scan (n = 6)	100	100	100	100

PPV: Positive predictive value in percentage.

NPV: Negative predictive value in percentage.

**Figure 3:**
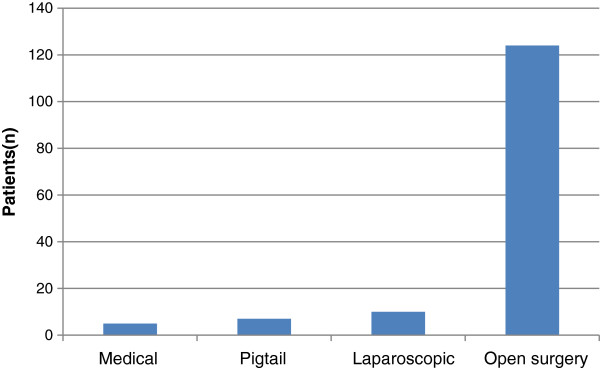
Hospital management.

The majority of our patients (89%, 131/146) were operated on soon after admission (Figure 3). Histology results showed perforated appendix with or without generalized peritonitis in 41 patients (29%) and normal appendix in 11% of cases (Table 2).

**Table 2:** Histological findings

**Histology findings**	**Number (%)**
Perforated appendix/generalized peritonitis	41 (28.7)
Gangrenous appendicitis	9 (6.6)
Inflamed appendix	38 (26)
Normal appendix	16 (10.9)
Missing	42 (28.7)

The mortality rate was 1.37% (2/146); patients who died were above 45 years of age, with comorbidities and having had more than two re-operations. There was a statistically significant difference in duration of symptoms, length of ICU and hospital stay, re-operation, and mortality in patients with complicated appendicitis when compared to uncomplicated appendicitis (Tables 3 and 4).

**Table 3:** Comparison of complicated and uncomplicated appendicitis

**Parameter**	**Uncomplicated number (%)**	**Complicated number (%)**	***P *value**
Male	56 (55.45)	35 (77.78)	0.01
Female	45 (44.55)	10 (22.22)	
Average age ± SD	26 ± 12	25 ± 13	0.791
Duration of symptoms			<0.001
<2 days	39 (38.61)	2 (4.44)	
>2 days	62 (61.39)	43 (95.56)	
Previous GP treatment	12 (42.86)	16 (57.14)	<0.001
Temperature			0.514
<37.5°C	87 (70.16)	8 (36.36)	
>37.5°C	14 (63.64)	8 (36.36)	
WCC			0.102
<12x10^9^/L	49 (35)	25 (18)	
>12x10^9^/L	39 (28)	25 (18)	
CRP			0.06
<10 mg/L	13 (14)	1 (3)	
>10 mg/L	79 (86)	36 (97)	
ICU admission			<0.001
<2 days	5 (4.95)	9 (20)	
>2 days	1 (0.99)	11 (24.44)	
Hospital stay			<0.001
<2 days	39 (38.61)	2 (4.44)	
>2 days	62 (61.39)	43 (95.56)	
Mortality	0 (0.00)	2 (1.37)	<0.001

The *P* values for categorical variables were derived from two-tailed χ^2^ test or Fisher’s exact test; *P* values for continuous variable were derived from Student’s *t*-test.

**Table 4:** Comparison of findings in complicated appendicitis

**Parameter**	**Inflammatory mass number (%)**	**Appendix abscess number (%)**	**Generalized peritonitis number (%)**	***P *value**
Male	2 (50)	7 (87.5)	26 (78.78)	0.004
Female	2 (50)	1 (12.5)	7 (21.22)	
Average age ± SD	27 ± 16	20 ± 10	25.8 ± 13	0.777
Duration of symptoms				0.049
<2 days	1 (1.33)	3 (4)	14 (18.67)	
>2 days	3 (4.73)	5 (7.04)	25 (33.80)	
Previous GP treatment	1 (3.75)	3 (10.71)	13 (46.43)	0.008
Temperature				0.92
<37.5°C	4 (100)	7 (87.5)	31 (81.58)	
>37.5°C	0 (0)	1 (12.5)	7 (18.42)	
WCC				0.160
<12x10^9^/L	3 (4)	3 (4)	15 (20)	
>12x10^9^/L	0 (0)	4 (6.35)	20 (31.75)	
CRP				0.003
<10 mg/L	1 (7.14)	0 (0.00)	0 (0.00)	
>10 mg/L	2 (1.74 )	7 (6.09)	31 (26.96)	
ICU admission				<0.001
<2 days	1 (2.44)	0 (0.00)	1 (2.44)	
>2 days	3 (2.74)	8 (7.63)	37 (35.5)	
Hospital stay				<0.001
<2 days	0 (0.00)	3 (21.43)	8 (57.14)	
>2 days	0 (0.00)	2 (16.69)	9 (75)	
Mortality	0 (0.00)	0 (0.00)	2 (1.37)	<0.001

The *P* values for categorical variables were derived from two-tailed χ^2^ test or Fisher’s exact test; *P* values for continuous variables were derived from Student’s *t*-test.

## Discussion

Our study involved 146 patients out of a total of 3,994 patients admitted during a six-month period to the Department of Surgery at CHBAH. Signs and symptoms of acute appendicitis were dominated by abdominal pain felt in the RIF in 95% of patients, vomiting in 73%, and nausea in 80%, while the typical clinical presentation as described in the standard textbooks was found in 31% of the 146 studied patients. The overall complicated appendicitis rate was 31%. Walker and Segal found that 210 patients out of a total of 24,000 surgical admissions presented with acute appendicitis at CHBAH, while Murchison Hospital, in Southern Kwazulu Natal, reported only seven cases of acute appendicitis out of 8,000 admissions, with a potential population draining of 200,000 people to this particular hospital [9]. At Frere Hospital, Rogers et al. estimated acute appendicitis at 17 admissions per month in 2006 [10]. We estimate the current average in our hospital (CHBAH) at 25 cases per month. In the literature, the peak incidence of acute appendicitis worldwide is between 10 and 30 years of age [1]. In agreement with this, our study shows that acute appendicitis is common in young adults with an average age of 26 years (SD = 12 years); 62% (91/146) of patients included in our study were male, which confirms previous findings that 67% (143/212) and 33% (69/212) of patients presented with acute appendicitis to CHBAH were male and female, respectively [11]. Indeed, our study shows a statistically significant difference in the occurrence of complicated appendicitis regarding gender (Table 4). Most importantly, this finding further confirms the predominance of acute appendicitis in young males.

The average duration of symptoms in our study was 4.5 ± 4 days. Compared to other studies, the average duration of symptoms before seeking medical attention was high, which might explain the heightened rate of complicated appendicitis found in our study. Victor et al. found that the mean duration of illness prior to seeking medical attention was 3.7 days, while Chamisa, at Prince Mshiyeni Memorial Hospital, found delays of 4 ± 3.5 days in presentation [12,13]. Importantly, our study confirms a statistically significant difference in patients with uncomplicated and complicated appendicitis after two days of symptoms (*P* <0.001). Indeed, our finding is in agreement with various studies showing that the rate of complicated appendicitis increased two days after onset of symptoms [2,8,14]. Hayden et al*.* reported the risk of perforation at 70% after 48 hours of symptom onset [14]. Eldar et al*.* showed that the risk of perforation is minimal before 36 hours after onset of symptoms, but increases thereafter [15].

The present study included all the standard different investigations required in the diagnosis of acute appendicitis cases. We found the inflammatory marker, CRP, sensitive in up to 92% of cases and WCC in 48%, with NPVs of CRP and WCC being 50% and 28%, respectively. Ahmad et al. found the CRP sensitivity to be 93% and the specificity 86%, while the total leukocyte count had a NPV of 50% and CRP had a NPV of 50% [16]. Bearing in mind that ultrasound is operator-dependent, we found sensitivity to be 60%, specificity 66%, PPV 86.9%, and NPV 31%. In contrast, Al-Ajerami found an ultrasound sensitivity of 84.8% and a specificity of 83.3%, with a PPV and a NPV of 93.3% and 66.7%, respectively [6]. In general, ultrasound seems to have better PPV than NVP. Our study shows, as many previous studies have shown, that CT scanning is the best method of investigation to confirm or to invalidate the diagnosis of appendicitis [17,18].

Our study shows that 63% of patients presented with delays, with the major reason for delay being lack of disease awareness and health facilities. Of those who presented late, 30% had self-medicated; 19% of the delayed presentations had been treated previously by general practitioners and most of these patients had been put on antibiotics. Thirty percent of acute appendicitis cases in our study were complicated appendicitis. Levy et al., in their audit of 1997, found the rate of perforation at CHBAH to be 22%. Madiba et al., at King Edward VIII Hospital in Durban, showed a perforated appendicitis rate of 34% and has associated this with delayed presentation [19]. Victor et al., at Edendale Hospital, found perforation of appendix cases to be 57% (114/200), of which 19% (38/200) were referred from the surrounding primary healthcare clinics and 2.5% (5/200) were referred from local general practitioners [13]; referrals from the four rural referral hospitals constituted 35% (70/200) of admissions. In our study we found a lower rate of perforation compared to that of other hospitals in South Africa, such as Edendale Hospital and Frere Hospital [10,12,13]. Our study shows that 83% of all admissions underwent surgery. In their trial of treating acute appendicitis with antibiotics, Vons et al. found that 12% of patients on anti-biotherapy underwent appendicectomy during the first 30 days, while 30% underwent appendicectomy between 1 month and 1 year later [20]. Hanson et al. found that 23% of appendicectomies take place after a failure to initial anti-biotherapy [21]. The treatment of acute appendicitis with antibiotics requires specific protocols and thorough follow-up of the patients.

Our study shows that outcome strongly depends on the presentation of acute appendicitis (uncomplicated or complicated), the age at presentation, the duration of symptoms, re-operations, and ICU stays of more than two days, and that hospital stays of longer than two days in complicated appendicitis were significant compared to cases of uncomplicated appendicitis. This was also found in other studies which assessed the outcome in cases of acute appendicitis [12,13]. In our study, the overall mortality rate is 2/146 (1.37%); patients who died were above 45 years of age. Our mortality rate was acceptable compared to acceptable mortality rate of <1%. Similarly, Chamisa, at Prince Mshiyeni Memorial Hospital, reported a mortality of 1.2%, with all cases from the perforated group [12] and Victor et al., at Edendale Hospital, reported an overall mortality rate of 2% [13]. All of the patients who died in the study by Victor et al. had intra-abdominal contamination in all four quadrants and all patients required initial ICU admission [13].

Furthermore, our study shows that elderly patients who contract acute appendicitis have an atypical clinical presentation, most often with associated co-morbidities such as diabetes and hypertension. For this reason, the elderly patient requires particular attention: the correct diagnosis to be made as soon as possible and accurate investigations being essential if there is any doubt in the diagnosis of possible appendicitis.

## Conclusions

Patients with acute appendicitis rarely present with a typical history of vague peri-umbilical pain. Leukocyte count is not reliable in the diagnosis of acute appendicitis. Most of our patients present late, with complicated diseases, and the most common reason for delay in presentation being a lack of disease awareness and/or health facilities and prior treatment by general practitioners. Complications were higher in males and the elderly.

## Abbreviations

CHBAH: Chris Hani Baragwanath Academic Hospital; CRP: C-reactive protein; ICU: Intensive care unit; NPV: Negative predictive value; PPV: Positive predictive value; RIF: Right iliac fossa; WCC: White cell count.

## Competing interests

The authors declare that they have no competing interests.

## Authors’ contributions

RN designed the study, participated in data collection, analysis of the data, has written the first manuscript. TE L and D K participated in Data collection and revised the manuscript. All authors contributed to the manuscript and approved the final version.
